# Letter to the editor — When something goes wrong

**Published:** 2019-12-17

**Figure:**
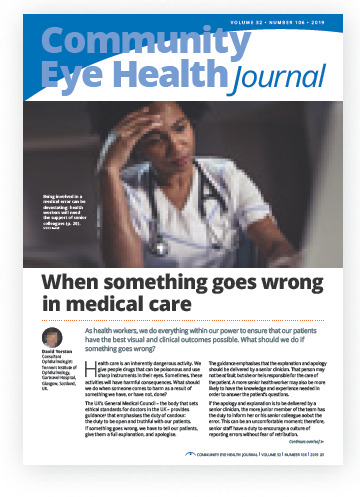


Thank you so much for your courageous coverage of medical error in the most recent issue of the *Community Eye Health Journal*. Inadvertent harm in health care settings can be devastating for patients and caregivers alike. Not too long ago, when I was trained in medicine, disclosure of medical error and apology were discouraged because of the potential for lawsuits. Such an approach disrespected patients and morally harmed caregivers. It was therefore tremendously encouraging to learn that, at least in clinical eye care, disclosure of error and apology are being practiced in hospitals and clinics around the world. A recent account in the Huffington Post by a gynaecologist (**http://bit.ly/Huff-apology**) complements your reporting and highlights the positive impact of disclosing medical error.

When something goes wrong in public health, or global health, offering an apology can be even more difficult. Responsibility is diffuse and causal pathways are more difficult to discern. There may be fear that acknowledging inadvertent harm could threaten public health programmes that deliver substantial benefits. Consequently, as described in a recent article (**http://bit.ly/glob-apol**), apology in public health is less often the norm. We in public health can be inspired and challenged by the progress made by eye health in acknowledging unintended harm.

Your remarkable coverage of this topic in the *Community Eye Health Journal* has done us all a great service. Indeed, this issue can serve as a model for other fields within health care and across global health. Thank you for so positively advancing the conversation, with extraordinary clarity and forthrightness.

## David Addiss

Director: Focus Area for Compassion and Ethics (FACE), Task Force for Global Health, Decatur, USA. Web: **www.taskforce.org** Email: **daddiss@taskforce.org**

